# Plant-derived products as anti-leishmanials which target mitochondria: a review

**DOI:** 10.1017/erm.2025.8

**Published:** 2025-03-26

**Authors:** Chandrima Shaha

**Affiliations:** Division of Infectious Diseases and Immunology, Indian Institute of Chemical Biology, Kolkata, India

**Keywords:** drugs, electron transport, Leishmaniasis, mitochondria, plant-derived products

## Abstract

**Background:**

The global incidences of leishmaniasis are increasing due to changing environmental conditions and growing poverty. Leishmaniasis, caused by the *Leishmania* parasite, presents itself in six different clinical forms, the cutaneous and the visceral diseases being the most prevalent. While the cutaneous form causes disfigurement, the visceral form could be fatal if not treated. With no available vaccines combined with serious side effects of current medications and emerging drug resistance, it is crucial to discover new drugs whether as novel compounds or as repurposed existing pharmaceuticals. In the realm of drug development, mitochondria are recognized as important pharmacological targets due to their critical role in energy control, which, when disrupted, leads to irreversible cell damage. Certain plant-based compounds able to target the parasite mitochondrion, have been studied for their potential anti-leishmanial effects.

**Search results:**

These compounds have shown promising effects in eliminating the *Leishmania* parasite. Artemisinin and chloroquine, two anti-malarial drugs that target mitochondria, exert strong anti-leishmanial effectiveness in both *in vitro* cultures and *in vivo* animal models. Quinolones, coumarins and quercetin are other compounds with leishmanicidal properties, which disrupt mitochondrial activity to effectively eliminate parasites in animal models of the disease and could be considered as potential drugs.

**Conclusions:**

Therefore, plant-based compounds hold promise as potential candidates for anti-leishmanial drug development.

## Introduction

A considerable section of the global population uses plant-based medicines to cure ailments, and about 75% of medications created to treat infectious diseases have their roots in nature (Refs 1–3). In light of the above findings, the search for medications derived from plants to treat neglected tropical illnesses continues. Recent technological advancements give access to a vast array of novel skeletons from plant sources where combinatorial modification may be able to yield suitable non-toxic products (Ref 4, 5). Leishmaniasis is one of the seven most serious tropical diseases with 700,000 to 1 million new cases reported annually (Ref 6). The current chemotherapy for leishmaniasis is not efficient due to long administration schedules, resistance development, high cost, clinical failures and toxicity, so the development of new treatments has been prioritized (Ref 7). To achieve the elimination of leishmaniasis, the discovery of new anti-leishmanial compounds or repurposing of existing drugs is necessary (Refs 8, 9). This focused review attempts to identify available plant-based compounds that target the parasite’s mitochondrion to disrupt cellular function and have been tested in the human or animal system.

## Search strategy

Electronic databases of PubMed and Google Scholar were used with ‘plants’ and ‘*Leishmania*’ as keywords for a period span of years 1985–2024. About 1718 results were listed. From this, the selection of articles was performed based on the title and subsequently the evaluation of the abstract for eligibility, followed by an assessment of the full text for relevance. From here, 160 references were extracted and further analyzed, according to the inclusion/study selection criteria for plant extracts having good anti-leishmanial effects affecting the mitochondria and already tested in humans or animals. This forms the primary body of the review. A separate section at the end evaluates some possible compounds that have been only tested *in vitro* but target the mitochondria of the parasite. This information may form a basis for future explorations in suitable models.

## Leishmaniasis and the *Leishmania* parasite

**Figure 1.**
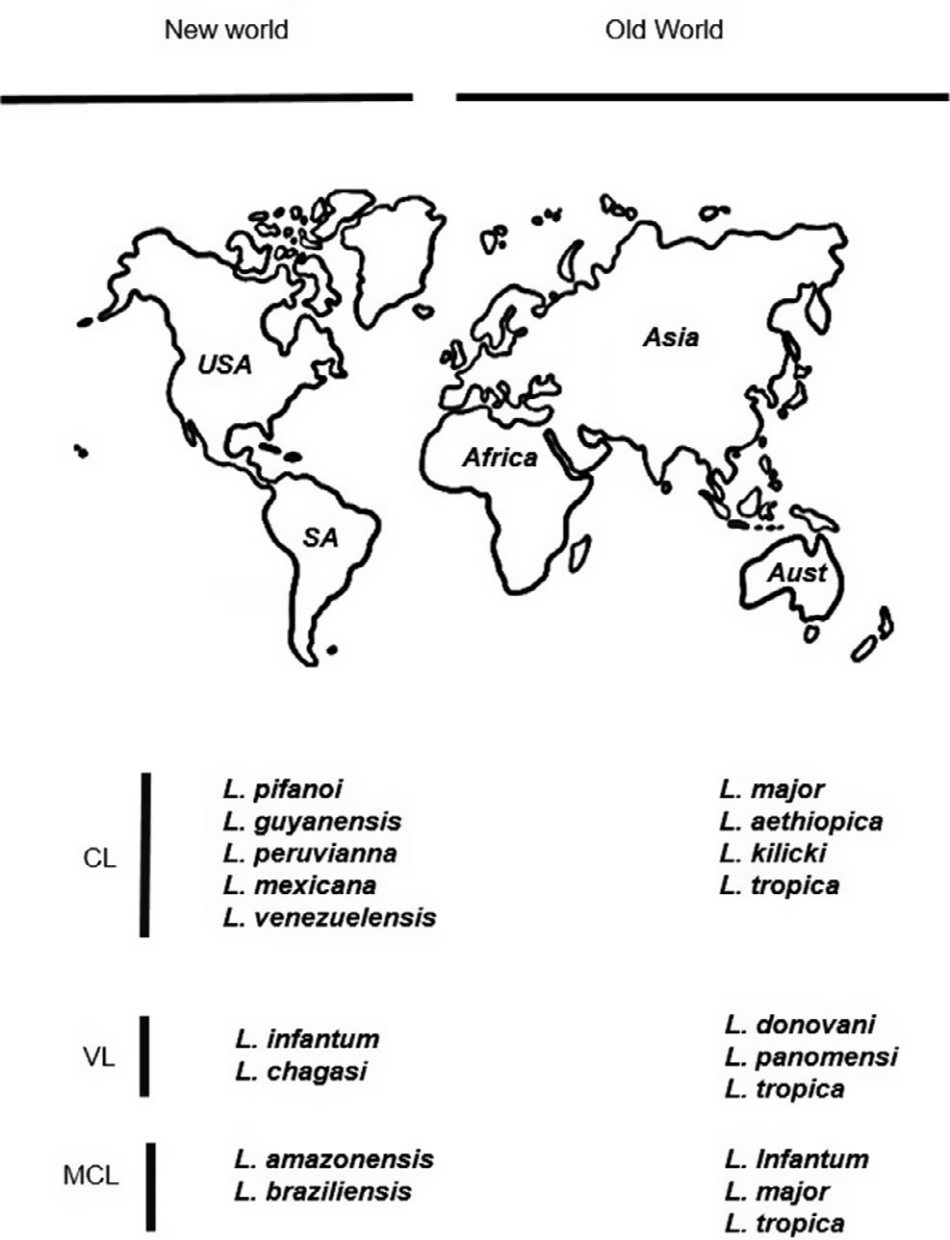
Distribution of leishmaniasis in the Old World and the New World. The figure shows species distribution of the *Leishmania* parasite in the old world and the new world (Ref 16). CL: cutaneous leishmaniasis; MCL: mucocutaneous leishmaniasis; VL: visceral leishmaniasis.

Leishmaniasis, caused by the parasite *Leishmania* manifests in six clinical forms, namely, visceral leishmaniasis (VL), post-kala-azar dermal leishmaniasis (PKDL), cutaneous leishmaniasis (CL), diffuse cutaneous leishmaniasis (DCL), mucocutaneous leishmaniasis (MCL) and mucosal leishmaniasis (ML)(Ref 10). Over the past few years, zoonotic or anthroponotic transmissions have caused the re-emergence of VL and CL because of political instability, deforestation and climate change (Refs 11–13). VL is the second most lethal parasitic disease after malaria and is fatal in the absence of therapy (Ref 14). VL can also present as PKDL, a cutaneous variant of the illness, which presents a risk of recurrence because people with PKDL act as reservoirs for the parasite that causes VL (Ref 15). Different species of the parasite are distributed all over the world and for reference are designated as the Old World species and the New World species (Ref 16) (Figure 1).

Chemotherapy with pentavalent antimonials as sodium stibogluconate, has been used as a first-line drug against VL since 1945 and is still being used for the treatment of canine leishmaniasis (Ref 17). The use of antimony in humans is limited because of the development of resistance, high levels of toxicity and occasionally therapeutic failure (Ref 18). Amphotericin B (AmB), a polyene antibiotic is used as an anti-leishmanial which is more effective in lipidic presentations (Ref 18) but high cost and toxicity prevent its widespread use. Oral drug Miltefosine is a neoplastic agent that has been used as an anti-leishmanial but teratogenic effects have limited its use (Ref 19). An aminoglycoside antibiotic, paromomycin, was also employed as an anti-leishmanial medication, but its usage was restricted due to the quick emergence of resistance (Ref 20). Pentamidine showed high toxicity when used against VL and is now used mostly for CL (Ref 18).

**Figure 2.**
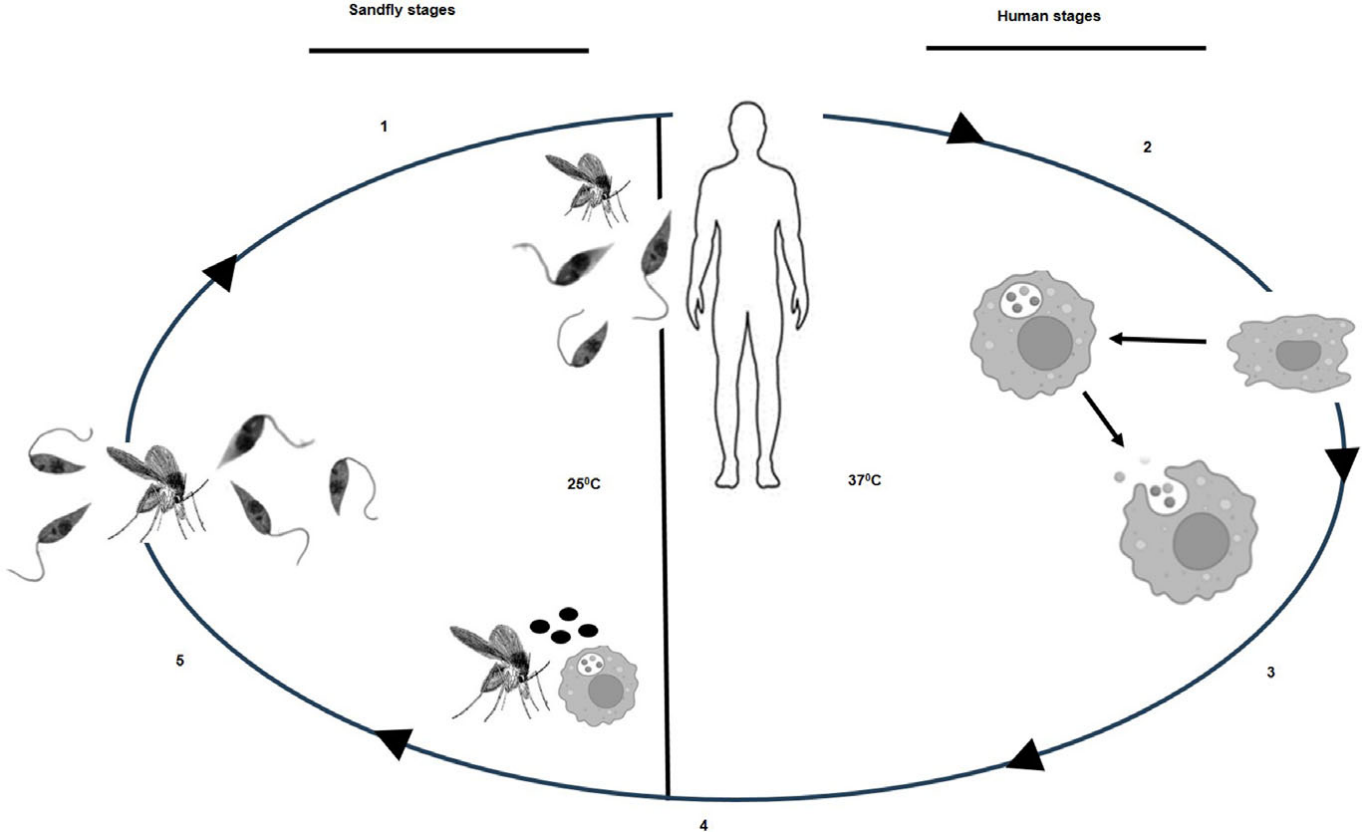
Life cycle of the *Leishmania* parasite. (1) Sandfly, the invertebrate host, releases infective metacyclic promastigotes in the mammalian bloodstream while taking a blood meal. The parasites enter an environment of 37°C in the mammalian skin from where they are picked up by the phagocytic cells. (2) The promastigotes convert to amastigotes and proliferate within the phagolysosomes of the mammalian macrophages. (3) Amastigotes are released from the macrophages when saturating numbers are reached within a cell. (4) Amastigotes and parasitized macrophages are picked up by the sandfly during a bite. (5) Amastigotes convert to promastigotes within the gut of the sandfly and the infective metacyclic forms move to the proboscis to be delivered to the mammalian bloodstream during a bite (Ref 14).

The *Leishmania* parasite exhibits two morphological forms during its digenetic life cycle in two different hosts, a mammalian host and an invertebrate host the sand fly (Figure 2). Humans, dogs and rats are the mammalian hosts, while the invertebrate host, the sand-fly belongs to either the *Phlebotomus* or *Lutzomyia* genus. Parasites picked up from infected mammals grow in the sand fly gut as free-swimming forms (promastigotes) at a temperature of about 25^°^C and upon maturity move to the proboscis of the fly as infective metacyclic promastigotes (Ref 14). These free-swimming metacyclics are introduced into the skin of the mammalian host (37°C) through the sand fly bite, from where they are picked up by the circulating phagocytes for elimination. Eventually, the parasites that survive the host defense processes, establish themselves within the phagolysosomes of macrophages, after undergoing major changes in morphology as stationary amastigotes (Ref 21). These amastigotes are the disease-causing forms.

## Models for testing of anti-leishmanial agents

### *In vitro* cultures for testing anti-leishmanial compounds

Anti-leishmanial drugs are tested *in vitro,* using both the intracellular amastigotes and the free-swimming promastigotes. For culturing *Leishmania* parasites from cutaneous leishmaniasis patients, parasites are recovered from a needle biopsy sample of the skin of an infected person, and after purification, parasites are put in culture either in blood agar slants or in enriched media (Ref 22). For collecting specimens for culture for the parasites causing the visceral form of the disease, bone marrow, spleen, liver, lymph node or other tissue biopsies are taken and put in culture either in blood agar slants or in enriched media. After incubation, parasites are retrieved (Ref 23).

The promastigotes divide rapidly *in vitro* in the logarithmic phase which transforms into stationary phases from late log phase promastigotes and are highly infective (Ref 24). Amastigotes obtained from human biopsies or infected animals or macrophages infected *in vitro* (Ref 25) are used for experiments. Axenic amastigotes, a type of amastigote commonly employed in *in vitro* research, are generated from promastigotes cultivated at 37°C and pH 5.0, which replicates the conditions of a phagolysosome of the host macrophage that the parasite uses as the survival niche (Ref 26).

Typically, three methods are employed for *in vitro* experiments with amastigotes. First, mammalian macrophage cultures are infected with promastigotes of *Leishmania* spp. These parasites will transform into amastigotes between 3 and 5 days (depending on the species of the parasite). The parasites are then collected from the macrophages by differential centrifugation (30 *g* for macrophages and 700 *g* for the parasites). The second technique is the use of axenic amastigotes *in vitro*, generated by the method stated in the earlier paragraph. The third possible way is to collect amastigotes from skin lesions or liver biopsies and use them for *in vitro* testing.

### *In vivo* testing for anti-leishmanial compounds

For *in vivo* animal testing, mice or hamsters are the most common models while dog and non-human primates are used less frequently (Ref 27). Several *Leishmania* species like *Leishmania major (L. major*)*, Leishmania mexicana* (*L. Mexicana)* and *Leishmania amazonensis* (*L. amazonensis)* are used to generate CL (Ref 27) by injection in the foot pad of the animal. Sensitive BALB/c mice get extensive lesions that lead to the progression of the illness and serve as models for the study of CL (Ref 27).

For generating VL, mice are challenged intravenously with late log or stationary phase promastigotes of *Leishmania infantum* (*L. infantum)* or *Leishmania donovani* (*L. donovani).* Two to 4 weeks after injection, amastigotes can be recovered from the spleen and liver (Ref 28). The most suitable model for visceralizing *Leishmania* infections is the Syrian golden hamster (*Mesocricetus auratus*) (Ref 28). A canine model and the Asian rhesus macaques (*Macaca mulatta*) are quite susceptible to the *Leishmania* infection and are used as disease models (Ref 27).

## The *Leishmania* mitochondrion and the electron transport chain

There are prominent differences between the *Leishmania* mitochondrion and the mammalian mitochondria. In terms of numbers, there is a single long-branching mitochondrion in the *Leishmania* cells that extends throughout the body (Ref 29). In contrast, there are smaller mitochondria in large numbers that populate the mammalian cells. In the parasite, this organelle contains a dense complex of DNA near the basal body towards the flagellar end called the kinetoplast which is not present in mammalian mitochondria. Unlike the mammalian mitochondrial DNA, the *Leishmania* mitochondrial DNA (mtDNA) is made up of thousands of minicircles and some maxicircles that form a complex network (Ref 30). The mitochondrial electron transport chain is an important target as interference with its function impacts the survival of the parasite. The cytochrome bc1 complex is one such target. In addition, the protein-importing machinery of the *Leishmania* mitochondria is a possible target, like the specific protein pATOM36 (peripheral atom 36), the archaic translocase of the outer mitochondrial membrane is not present in mammalian cells (Ref 31). In addition, defense proteins like the mitochondrial tryparedoxin peroxidase in the mitochondria, unique to the parasite would be specific targets.

Existing drugs like amphotericin B, miltefosine, pentamidine and antimonials interfere with the mitochondrial membrane permeability of the parasite, resulting in a collapse of the mitochondrial membrane potential (ΔΨm) (Refs 32–34). Given the susceptibility of the parasite mitochondria to external agents, it is a good drug target. In general, mitochondria are recognized as targets for drugs (Ref 35) because they control the most important aspects of cell viability like the energy balance, essential for homeostasis and survival. Since any interference with energy homeostasis would be an ideal target, in the last decade, many mitochondria-targeted drugs have been synthesized and tested in the mammalian system and some are in clinical trials (Ref 36–38). These drugs, already found effective in the mammalian system, should be tested as anti-leishmanial agents in both *in vitro* and *in vivo studies.* In this review, compounds affecting the parasite mitochondria have been highlighted with the expectation that this information will serve as a base for designing new molecules or repurposing existing drugs.

**Figure 3.**
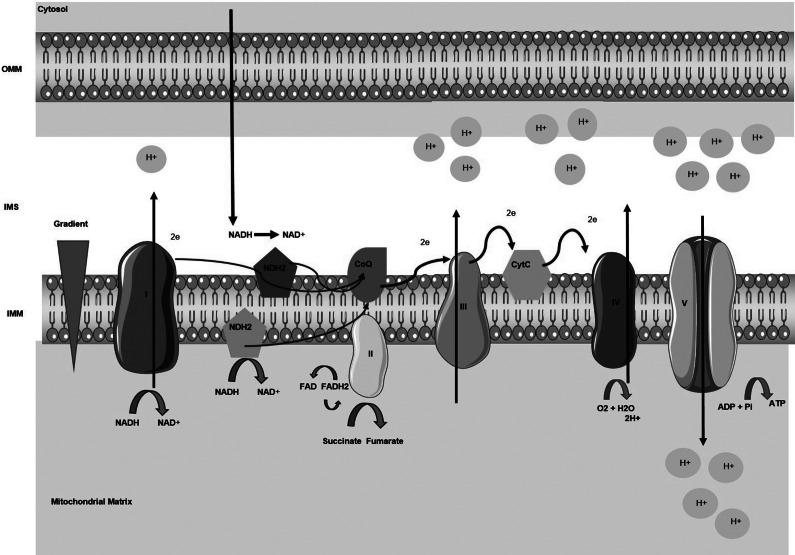
The electron transport chain. Complex I (NADH ubiquinone oxidoreductase), complex II (succinate ubiquinone oxidoreductase); complex III (cytochrome c3+ oxidoreductase); complex IV (cytochrome C oxidase with ubiquinone) are located on the inner mitochondrial membrane as integral membrane proteins. Complex I transfers two electrons to ubiquinone when a simultaneous translocation of protons occurs. Coenzyme Q and cytochrome C serve as mobile electron carriers to facilitate the production of ATP through oxidative phosphorylation. CoQ10 allows the transfer of electrons to complex III which transfers these electrons to the cytochrome C responsible for connecting to complex IV where the reduction of O_2_ to H_2_O will take place. Complexes I, III, and IV function as proton pumps. The generation of an electrochemical gradient between the intermembrane space and the mitochondrial matrix occurs because of the pumping of protons. The ΔΨm thus produced drives ATP synthesis from ADP and inorganic phosphate by the F1F0 ATP synthase (Refs 35, 36). Cyt C: cytochrome c; IMM: inner mitochondrial membrane; IMS: Intermembrane space; OMM: Outer mitochondrial membrane; NADH: nicotinamide adenine dinucleotide + hydrogen.

The mitochondrial respiratory chain of parasites shows greater diversity in the electron transport complexes (ETC) than their hosts making them good targets for therapy (Ref 39). The ETC consists of integral membrane proteins like Complex I (NADH ubiquinone oxidoreductase), Complex II (succinate ubiquinone oxidoreductase), Complex III (cytochrome c3+ oxidoreductase) and Complex IV (cytochrome C oxidase with ubiquinone) localized on the inner mitochondrial membrane (Figure 3). The equilibrium of NADH+/NAD is preserved by the ETC, which is essential for cell viability. Complexes I, III and IV function as proton pumps by allowing protons to move from the matrix into the intermembrane space in opposition to the gradient (Figure 3). The pumping of protons results in the generation of an electrochemical gradient between the intermembrane space and the mitochondrial matrix. The ΔΨm thus produced, drives ATP synthesis from ADP and inorganic phosphate by the F1F0 ATP synthase (Ref 39). The ΔΨm is the main component of the electrochemical H^+^ gradient and is a dynamic measure of mitochondrial function. A break in the electron transport chain’s operation, ATP depletion, loss of ΔΨm, ROS generation or an increase in intracellular or mitochondrial Ca^2+^ could all lead to the functional disruption of mitochondria (Ref 40). The ΔΨm plays a central role in mediating the apoptotic process (Ref 41), but the alteration of ΔΨm can also occur secondary to (i) inhibition of the ETC, (ii) stimulation of uncoupling proteins and/or (iii) permeabilization of the inner membrane. Blocking the ETC can generate toxic reactive oxygen species (ROS) such as superoxide anion (O2^−^), and uncoupling of the ETC in kinetoplastid parasites was shown to increase the generation of toxic ROS, with complex III, also known as cytochrome bc1, being the principal ROS source (Ref 41). Therefore, the delivery of drugs to the mitochondrion of *Leishmania* could be designed to inhibit the mitochondrial permeability transition (MPT), uncouple the electron transport chain (ETC) or activate the uncoupling proteins.

The *Leishmania* parasite while infecting the host, faces severe oxidative stress from the cells of the host immune system. Particularly, it is a disadvantage that the parasite lacks catalase which is essential to eliminate reactive oxygen species, and therefore, *Leishmania* has adapted to use a different enzyme system, the tryperodoxin peroxidases. These enzymes are located in the cytosol and the mitochondria and are used by the parasite to eliminate hydrogen peroxide and products generated by it (Ref 42).

## Plant-derived natural products as anti-leishmanial agents targeting the mitochondrion

### Artemisinin

**Figure 4.**
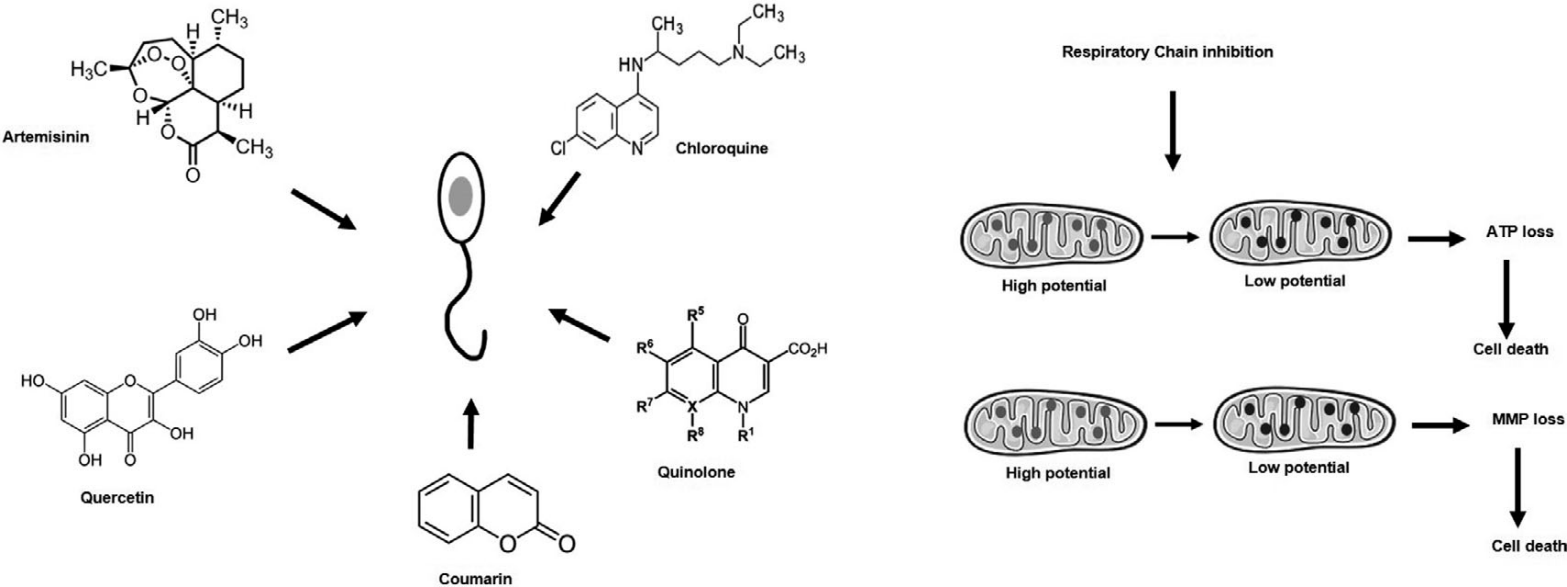
*In vivo* tested plant-based possible anti-leishmanials that could target mitochondria. Artemisinin, chloroquine, quercetin, coumarin and R1–R5 quinolone are shown.

Artemisinin, a sesquiterpene lactone, and its derivatives have been widely used for malaria. Based on references to the use of the herb *Artemisia annua* in Chinese traditional medicine, artemisinin was discovered by Tu Youyou in 1972 (Ref 43). Through a collaborative effort, Chinese scientists prepared dihydroartemisinin, artemether and artesunate generically known as ‘artemisinins’ from *Artemisia annua* that were used as anti-malarial medications (Ref 43). WHO recommends artemisinin-based combination therapies as the second line of treatment for *P. vivax* that are resistant to chloroquine and the first line of treatment for uncomplicated *P. falciparum* malaria (Ref 44). As a remedy for malaria in many endemic areas, artemisinin derivatives such as artesunate and artether are used solo or in combination (Ref 45, 46). Artemisinin is a tetracyclic 1,2,4-trioxane containing an endoperoxide bridge (C–O–O–C), the key pharmacophore of the drug. (Figure 4). Notably, it is not only malaria that artemisinin is used for, but it is also prescribed for phylogenetically unrelated parasitic infections of public health importance like schistosomiasis (Ref 47) or other helminthic diseases caused by nematodes, trematodes, cestodes and others (Ref 48–50). Therefore, this drug has a wide range of efficacy against parasites which makes it suitable for repurposing.

In an *in vitro* and an *ex-vivo* study for VL, apoptotic death of *L. donovani* promastigotes and intracellular amastigotes was induced by artemisinin with IC_50_ values of 160 and 22 μM, respectively (Ref 51). A loss of mitochondrial potential occurred before initiation of the death process (Ref 51). Other studies confirmed similar observations in *in vitro* and *ex-vivo* studies showing leishmanicidal activity against *L. donovani* with 50 % inhibitory concentration of 14.63 ± 1.49 μg ml^−1^ and 7.3 ± 1.85 μg ml^−1^, respectively, against the promastigotes and intracellular amastigotes. Reactive oxygen species (ROS) and nitric oxide NO were shown to precipitate mitochondrial changes leading to parasite death by apoptosis (Ref 52). The leishmanicidal activity shown in *in vitro* and *ex-vivo* experiments was supported by *in vivo* studies in a model of leishmaniasis in infected BALB/c mice where the extracts were 90% effective in eliminating parasites (Ref 52). Artemisinin incorporated in liposomes with increased availability demonstrated a noteworthy anti-leishmanial efficacy both *in vivo* in *L. donovani*-infected BALB/c mice and *ex vivo* in murine macrophages (Ref 53, 54). In the BALB/c mice, the efficacy was excellent with percentage inhibition of the parasite load of 82.4% ± 3.8% in the liver and 77.6% ± 5.5% in the spleen (Ref 54). Already tested in humans for safety (Ref 55), artemisinin and its derivatives or the combinations could be considered as potential anti-leishmanials.

For CL, intracellular amastigotes of *L. amazonensis*, prevalent in the Brazilian Amazon region and capable of causing CL and MCL, were eliminated when exposed to artemisinin, artemether, artesunate and dihydroartemisinin (Ref 56). The *in vitro* studies were supported by *in vivo* observations of CL in BALB/c mice created by *L. amazonensis* where artemisinin and artesunate could reduce parasite load and lesion size (Ref 56). Artesunate, a more stable artemisinin derivative effective in eliminating *L. amazonensis* parasites, also reduced lesion size and associated inflammation (Ref 57). *L. major*, another species of *Leishmania* parasite causing CL was also eliminated from skin lesions (Ref 58). The efficacy shown *in vivo* generates possibilities for artemisinin and its derivatives as possible drugs for CL as well.

Mechanistically, artemisinin inhibits complexes I–III and II–III of the *Leishmania* ETC *in vitro* rapidly increasing ROS, thus disrupting mitochondrial function (Ref 59). The endoperoxide 1,2,4-trioxane ring in artemisinin is responsible for the action of artemisinin against malaria (Ref 60). Structurally, the unusual peroxide bridge of artemisinin in the presence of reducing agents such as Fe^2+^, haem and Cu^2+^ becomes unstable by cleavage, a cause of the generation of carbon and oxygen-centred free radicals (Ref 61). The suppression of ETC is a major source of ROS (Ref 62) especially superoxide. The resulting mitochondrial malfunction affects the energy needs of *Leishmania* parasites, with ATP depletion causing an energy catastrophe that ultimately results in death (Ref 59). Data on mitochondrial ΔΨm in the *Leishmania* parasite post artemisinin treatment corroborates observations of the collapse of the ΔΨm in malaria parasites (Ref 63). The respiratory control rate which is a measure of functioning of the mitochondrial ETC, is reduced by 30% when dihydroartemisinin is added to *L. braziliensis* cultures, suggesting a mitochondria-targeted action (Ref 64). The above studies give a clear indication that the primary site of artemisinin action in the parasite is the mitochondria and with its anti-leishmanial efficacy could be considered for therapy against leishmaniasis.

### Chloroquine

Encouraged by the scaffold of quinine, chloroquine, an alkaloid, was first synthesized by chemists in 1934 in Germany (Ref 65, 66). It is an analog of quinoline with an aliphatic amino side chain at the 4-position and a chloro group at the 7-position (Ref 66)(Figure 4). Chloroquine was the drug of choice for malaria till new antimalarials like artemisinin, mefloquine and pyrimethamine were developed. Since then, chloroquine and its derivatives have been repurposed for HIV, systemic lupus erythematosus, rheumatoid arthritis and several other diseases (Ref 67, 68) and are being tested for anti-tumor therapies based on their action on the autophagic activities of the cell (Ref 69). The distribution of its range of action prompted scientists to test chloroquine and its derivatives for anti-leishmanial efficacy.

Quinolinyl β-enaminone derivatives showed leishmanicidal activity against *L. donovani* by impairing the mitochondrial ETC complex and inducing ROS-mediated programmed cell death with 84% clearance of parasites in infected mice and hamsters (Ref 70). The addition of small heterocyclic rings on the pendent nitrogen of the 4-amino-7-cholroquinoline with replacement of the 7-chloro group with a trifluoromethyl group showed 81-99% inhibition at 10 μM concentration of promastigotes in *in vitro* cultures. The majority of the analogs demonstrated *in vitro* anti-amastigote action (IC_50_ = 5.3–9.6 μM). The substances that demonstrated anti-amastigote activity *in vitro* were then examined *in vivo* on a hamster model of VL generated by *L. donovani.* At 27 days after infection, the 2,6-trichlorophenylthiazolidin-4-one molecule outperformed others, showing around 80% elimination of the *Leishmania* parasite in treated hamsters (Ref 71). One benefit of these compounds is that they are well-tolerated by humans and the thiazolidin/thiazinan-4-ones are biologically advantageous scaffolds (Ref 72). Other studies reported the identification of two 4-amino-7-chloroquinoline compounds with adamantane or benzothiophene moieties being highly effective as anti-leishmanial candidates in a mouse model of VL using *L. infantum.* Parasite clearance from the liver was seen at 95% and 99% for doses of 50 and 100 mg/kg, respectively (Ref 73). Interestingly, Sitamaquin, an 8-aminoquinoline analogue, demonstrated superior anti-leishmanial effectiveness *in vivo* (Ref 74). This orally administered 8-amino-quinoline showed efficacy in various animal models of leishmaniasis and encouraging efficacy against various species of *Leishmania* was found in preliminary clinical studies in Kenya and Brazil (Ref 75). Nitroimidazo-oxazole compound DNDI-VL-2098 was previously identified as a favourable candidate but was found to cause organ toxicity in animal studies of longer duration and hence was not taken further (Ref 76). However, its oxazine derivative DNDI-0690 was recognized as a promising candidate for a Phase I trial for VL (Ref 77) and also for CL (Ref 78). The added advantage of the above compound is its efficacy against CL in addition to VL. In 2022, the evaluation of the safety and pharmacokinetic properties of DNDI-0690 was completed. The drug was advanced for consideration for efficacy testing in leishmaniasis patients (Ref 79). Therefore, the above compounds or their modified versions offer possibilities for treatment of VL.

The 4,7-dichloroquinoline linked together with diamines, diaminealkynes and diaminedialkynes, a series of 4-amino-7-chloroquinoline derivatives showed strong anti-leishmanial activity against *L. chagasi, L. braziliensis, L. major* and *L. amazonensis* all causing CL (Ref 67). These studies again suggested that modification at the 7-position is an attractive strategy for the development of anti-leishmanial compounds. Synthesized 4-aminoquinoline derivatives through conjugation of sulfonamide, hydrazide and hydrazine residues were effective in killing *L. amazonensis* promastigotes and intracellular amastigotes (Ref 80) showing their activity against CL. A chloroquine analog tested on *L. panamensis* causing tegumentary leishmaniasis showed good efficacy in an *in vivo* model of BALB/c mice but no mitochondrial interference was checked (Ref 81).

Mechanistically, aminoquinolines particularly target mitochondrial complex enzymes to disrupt the electron transport system in the mitochondria of protozoan parasites like *Plasmodium, Leishmania*, etc. (Ref 82). Sitamaquin inhibits complex II of the respiratory chain of *L. donovani* promastigotes (Ref 83) where oxidative stress is generated because the drug targets succinate dehydrogenase of complex II. Tafenoquine, an antiplasmodial 8-aminoquinoline, targets *Leishmania* respiratory Complex III and induces apoptosis (Ref 82). Exposure to 7-chloro-4-quinolinylhydrazone derivative to *Leishmania* induces a loss of mitochondrial potential and generates ROS leading to cell death (Ref 80). Chloroquine derivatives are also important disruptors of mitochondrial function through ATP inhibition because any interference with ETC would disrupt ATP (Ref 83).

### Coumarins

Coumarins are a naturally occurring class of phenolic compounds of the benzopyrone family first isolated from the plant named *Dipteryx odorata* Willd. by Vogel in 1820 (Ref 84). Coumarins have a wide variety of structures with coumarin 7 as the privileged scaffold (Ref 85). They are aromatic organic chemical compounds that could be described as having a benzene molecule with two adjacent hydrogen atoms replaced by an unsaturated lactone ring forming a 6-membered heterocycle that shares two carbons with the benzene ring (Figure 4). This bicyclic heterocycle is capable of performing interactions with various biological targets (Ref 82) and show a vast range of biological properties like anticoagulant, anticancer, antiviral, antitrypanosomal, antioxidant and also anti-leishmanial activities. Their biological activity as antimicrobials makes them attractive candidates for the development of antibacterial and antiprotozoal medicines. Coumarin derivatives are known to interfere with mitochondrial function and lately, several coumarins have been developed with mitochondrial target function that needs to be tested as anti-leishmanial agents (Ref 86). Coumarins have been used to treat prostate cancer, renal cell carcinoma, leukemia (Ref 84) and used as anticoagulants (Ref 87).

Studies conducted *in silico*, *in vitro* and *in vivo* demonstrate that coumarins are suitable candidates for assessment as anti-leishmanial medications (Ref 88). Five coumarins selected based on *in silico* studies were tested against CL. One derivative C2(6-Amino-3-(1,3-benzodioxol-5-yl)-2H-chromen-2-one) was used as nanoliposomal formulation against *L. major* promastigotes and significant anti-leishmanial activity was observed. There was a loss of ΔΨm after treatment with the compound in promastigotes. Continuing further with the study, testing in *in vivo* conditions showed a reduction in CL lesion sizes formed by *L. major* in BALB/c mice (Ref 88). Mammea A/BB, a coumarin isolated from leaves of *Calophyllum brasiliense* showed anti-leishmanial activity on both promastigotes and amastigotes of *L. amazonensis* with mitochondrial effects. Good *in vivo* activity was visible in the reduced size of skin lesions in the footpads of BALB/c mice (Ref 89, 90). Treatment with Mammea A/BB induced changes in the mitochondria of both promastigotes and amastigotes in terms of mitochondrial swelling with reduced density of the mitochondrial matrix and extra vesicles visible inside the mitochondrion, indicating damage and significant change in this organelle (Ref 90, 91). The details of any effect on the ETC are not known. Another coumarin derivative, Mammea A/BA showed IC_50_ of 85.8 and 36.9 μM for epimastigotes and trypomastigotes, respectively, of *Trypanosoma cruzi* a related parasite where a significant increase in ROS with decrease ΔΨm was observed (Ref 92). Subcutaneous treatment with (+)-3-(1′-dimethylallyl)-decursinol and (-)-heliettin, two coumarins extracted from *Helietta apiculata Benth* reduced size of cutaneous lesions generated in BALB/c mice with *L. amazonensis* by 95.6% and 98.6%, respectively (Ref 93). Osthole, a natural coumarin displayed dose-dependent leishmanicidal activity against intracellular amastigotes with IC_50_ value of 14.95 μg/ml. CL lesions generated in BALB/c mice with osthole resulted in declined lesion progression in comparison to suitable controls (Ref 94). Earlier, aurapten, a 7-geranyloxy-coumarin was shown to have anti-leishmanial activity (Ref 95). A review of natural and synthetic coumarins with anti-leishmanial efficacy details several important experiments and discusses the possibility of obtaining novel coumarin-based inhibitors (Ref 96). To identify the ETC complex component that coumarins affect, further study is required but coumarins could be considered as potential anti-leishmanials.

### Quinolones

Simple 3 carboxy quinolones were first described by J.R. Price and L.J. Drummond in 1949 (Ref 97). Quinoline alkaloids are secondary metabolites found in plants and are also produced by some bacterial species. Quinoline alkaloids have a wide range of biological activities including activity against cancer, malaria, inflammation and viruses (Ref 98, 99). Numerous quinolones like quinine, quinidine, cinchonine and cinchonidine have been obtained from natural sources since quinine was first extracted from the bark of the Cinchona plant in 1811 (Ref 100). Some of these have excellent medicinal properties and served as lead structures that provided the basis for synthetic quinolones as useful drugs (Ref 100). A few of these are very effective medications and were the model compounds from which synthetic quinolones were derived (Ref 100). Isolated from plant or microbial sources, several 4-quinolone alkaloids showed antimicrobial activities. The core structure is bicyclic (Figure 4; Ref 101). Endochin-like quinolones (ELQs) show promise as novel antiparasitic drugs, they are analogs of ubiquinone that can act as competitive inhibitors of cytochrome bc1 by binding to the Qi ubiquinone binding site (Ref 102, 103).

Studies show the efficacy of quinolones against CL caused by *L. amazonensis* and *L. venezuelensis* in infected BALB/c mice (Ref 104). Other quinoline alkaloids isolated from the *Galipea longiflora* Krause D and B act against *L. braziliensis* and *L. donovani* promastigotes (Ref 105). N-methyl-8-methoxyflindersin, isolated from the leaves of *Raputia heptaphylla* also known as 7-methoxy-2,2-dimethyl-2H,5H,6H-pyran[3,2-c]quinolin-5-one, shows anti-parasitic activity against *Leishmania* promastigotes and amastigotes (Ref 106). *In silico* tools used to identify synthetic quinoline alkaloids with similar structure to that of 7-methoxy-2,2-dimethyl-2H,5H,6H-pyran[3,2-c]quinolin-5-one tested for their *in vitro* antiparasitic activity against *Leishmania (Viannia) panamensis*, and *in vivo* therapeutic response in hamsters with experimental cutaneous leishmaniasis (CL) was reported (Ref 106). Endochin like quinolones deplete ATP in promastigotes and amastigotes but at a slow pace (Ref 102). Buparvaquone is a hydroxynaphthoquinone that is also related in structure to ubiquinone and is another potential inhibitor of cytochrome bc1 (Ref 102). This compound has demonstrated efficacy against *L. donovani in vitro* and in a BALB/c model of visceral leishmaniasis (Ref 107). Buparvaquone and its phosphate prodrugs (BPQ-3-PHOS and 3-POM-BPQ) were tested against several species of *Leishmania* (*L. major, L. amazonensis, L. aethiopica, L. mexicana* and *L. panamensis*) *in vitro.* BPQ, BPQ-3-PHOS and 3-POM-BPQ demonstrated low IC_50_ values against promastigotes and amastigotes of all *Leishmania* species tested (Ref 108). Buparvaquone is a potent inhibitor of electron transport and robustly depletes ATP at submicromolar concentrations within an hour *in vitro* resulting in amastigote death. Buparvaquone’s toxicity may stem from its capacity to enhance reactive oxygen species (ROS) production, possibly as a result of its inhibition of cytochrome bc1 activity (Ref 41). Buparvaquone significantly eliminates *L. donovani* parasites in mice models of leishmaniasis (Ref 107, 109). In an *in vivo* experiment, buparvaquone loaded with lipid nanoparticles with enhanced solubility demonstrates a 80% reduction in the livers’ burden of *L. infatum* (Ref 110, 111). In a different study, lipid nanoparticles coated with buparvaquone demonstrated 100% clearance of parasites in an *in vivo* model, whereas pure buparvaquone demonstrated only 50% efficiency in this regard (Ref 112). Cytochrome bc1 is thus a promising target for novel anti-leishmanial drugs, and further improvements on the buparvaquone scaffold are warranted for the development of enhanced therapeutics.

Quinolinyl β-enaminone derivatives showed anti-leishmanial activity through disruption of the mitochondrial ETC complex and induction of PCD after significant ROS production (Ref 66). Of the several compounds reported, the disruption of complex II by compound 3D ((Z)-1-(4-chlorophenyl)-3-(quinolin-8-ylamino)prop-2-en-1-one) was followed by oxidative burst, intracellular Ca^2+^ accumulation and activation of programmed cell death. *In silico* studies with compound 3D showed that mitochondrial complex II (SDH) or complex III (CCR) of *L. donovani*, modelled using the SWISS-Model server had more affinity towards SDH over CCR, which invariably correlates with its higher anti-leishmanial efficacy of 3D. This compound showed superior efficacy *in vivo* as well in infected BALB/c mice and hamsters (Ref 70) indicating this as a potential compound that can be tested for therapeutic purposes.

### Quercetin

Quercetin is a polyphenolic flavonoid with antioxidant characteristics found in a wide range of foods we consume, including citrus fruits and green leafy vegetables (Ref 113). It was first isolated in 1854 (Ref 114). In essence, quercetin is a pentahydroxyflavone with five hydroxy groups positioned at positions 3, 3, 3’, 4, 5 and 7 (Figure 4) and is known to possess anti-inflammatory, anti-proliferative, anti-oxidative, anti-diabetic, anti-carcinogenic and anti-viral properties. Several *in vivo* and *in vitro* studies demonstrate the role of quercetin in various diseases and a detailed review is available (Ref 115). The potential of quercetin as an anti-leishmanial agent was described in an early review (Ref 116). Following the release of the review, *in vitro* and *in vivo* investigations provided evidence supporting quercetin‘s potential as an anti-leishmanial agent. *L. amazonensis* and L. (V.) braziliensis, the causative agents of CL, *L. donovani* responsible for VL are sensitive to quercetin *in vitro* (Ref 117–119) and mitochondrial disruption is observed in both *L. amazonensis* and *L. (V.) braziliensis* along with ROS production (Ref 119, 120). *In vivo* studies in a murine model of CL created by *L. amazonensis*, a reduction in lesion size was observed (Ref 121). Effective against both *L. (V) brazilensis* promastigotes and intracellular amastigotes, quercetin showed anti-amastigote IC_50_ (21 ± 2.5 μM) and anti-promastigote IC_50_ (25 ± 0.7 μM) activities with a selectivity index of 22 (Ref 122). Quercetin was also effective as an oral drug in hamsters with CL (Ref 122, 123). Earlier studies with CL induced in BALB/c mice using *L. amazonensis* showed a reduction in lesion size with quercetin (Ref 121). Five weeks after inoculation, CL generated in BALB/c mice using *L. major* showed a dramatic reduction in lesion size following oral quercetin exposure for a sequential 28 days, (Ref 122). The anti-leishmanial impact of CL made with *L. amazonensis* in BALB/c mice was enhanced by the encapsulation of quercetin in lipid nanocapsules of poly(ϵ-caprolactone) (Ref 117). Quercetin may be taken into consideration for additional development given its effectiveness as an anti-leishmanial agent, as demonstrated by investigations both *in vitro* and *in vivo.*

Quercetin exerts its anti-leishmanial effect on *L. amazonensis* promastigotes through the disruption of mitochondria and generation of ROS (Ref 120). In mammalian cells, quercetin shows redox interaction with the mitochondrial ETC affecting its membrane potential and reducing ATP production. A review detailing interactions of quercetin with the ETC is available (Ref 124). Studies with *Leishmania* mitochondria need to be explored further to assess the effect of quercetin on mitochondria.

### *In vitro* tested plant compounds that affect the mitochondria

The following compounds have not been tested in higher animal models but are tested *in vitro* for efficacy with different *Leishmania* species and their effect on mitochondria has been analyzed. These could provide clues to taking up compounds for further studies for the development of anti-leishmanial agents. In *L. amazonensis* promastigotes, phytol-rich hexane fraction from the leaves of *Lacistema pubescens* induces depolarization of the mitochondria with an increase in ROS levels (Ref 125). Xanthohumol and resveratrol are naturally occurring constituents of the human diet. Resveratrol is formed as a phytoalexin in plants to defend against parasitic and fungal infections while xanthohumol belongs to the group of polyphenols. When tested with *Leishmania* parasites, both exerted anti-leishmanial action but in contrast to Resveratrol, xanthohumol strongly inhibited oxygen consumption in *Leishmania tarantolae* promastigotes. This was due to the inhibition of the mitochondrial electron transfer complex II/III by xanthohumol, which was less pronounced with Resveratrol (Ref 126). Essential oil from *Chenopodium ambrosioides* L. and caryophyllene oxide, a compound derived from the essential oil was able to inhibit the electron transport chain of *L. tarantolae* promastigotes depriving the parasite of energy sources (Ref 126). Iron can generate toxic ROS via the Fenton reaction. Studies to establish the influence of iron on the anti-leishmanial action of artemisinin *in vitro* showed that in Sneider’s insect medium, artemisinin caused a greater redox imbalance as compared to M199, a culture media where many experiments on *Leishmania* parasites grown *in vitro* are done (Ref 127). Therefore, the media type should be taken into account while analyzing the *Leishmania in vitro* tests. Several other compounds tested for anti-leishmanial effects through disruption of mitochondria primarily tested *in vitro* are described in a recent review (Ref 128). Berberine chloride is an alkaloid that shows anti-leishmanial effects where death is preceded by an upsurge in the generation of ROS along with a depletion of ATP. In addition, mitochondrial superoxide, depolarization of the mitochondrial membrane potential, dose-dependent inhibition of mitochondrial complexes I-III and II-III collectively suggest interference with mitochondria in *L. donovani* (Ref 129). Plumbagin, a naphthoquinone derivative results in the depolarization of the mitochondrial membrane, and depletion in ATP levels in the promastigotes of *L. donovani* inducing apoptosis-like death (Ref 130). Essential oil from *Chenopodium ambrosioides*, an aromatic herb, kills the parasite by causing a breakdown of mitochondrial membrane potential and a modification of redox indexes (Ref 35). Formulations of saponins and hydrazones cause changes in the ATP levels and induce depolarization of membrane potential with ROS increase (Ref 131). Ethanolic extract of *Croton blanchetianus* Ball is capable of causing depolarization of the mitochondria in *Leishmania amazonensis* promastigotes leading to death (Ref 132). Phloroglucinol derivatives from *Hypericum* species induce mitochondrial dysfunction like hyperpolarization and ROS generation in promastigotes of *L. amazonensis* promastigotes (Ref 133). A naturally occurring plant flavone Apigenin, shows anti-leishmanicidal action against *L. amazonensis* promastigotes through induction of swelling of the parasite mitochondria resulting in a loss of mitochondrial membrane potential with accompanying ROS increase (Ref 134). Melatonin, a neurohormone found in animals, plants and microbes used in humans as medication changes calcium homeostasis of the mitochondrion of *L. infantum*, with the opening of the mitochondrial permeability transition pore, loss of ATP and mitochondrial potential loss (Ref 135). Two plant-based compounds, piperine and phenylamide eliminate *Leishmania* promastigotes and amastigotes through induction of mitochondrial swelling and a loss of mitochondrial membrane potential (Ref 69). Caffeic acid shows anti-leishmanial activity against promastigotes and amastigotes of *Leishmania amazonensis* by the increase of oxidant activities (Ref 136). In mammalian cancer cells a variety of caffeic acid derivatives targeted to mitochondria have been developed (Ref 36). These should be tested in the *Leishmania* parasites both *in vitro* and *in vivo.* Curcumin (1,7-bis(4-hydroxy-3-methoxyphenyl)-1,6-heptadiene-3,5-dione) is a natural polyphenol found in the rhizomes of *Curcuma* spp. and is a well-known diferuloylmethane. Curcumin is a potent antioxidant agent with anti-inflammatory and anti-tumor properties. In several species of *Leishmania*, curcumin causes mitochondrial membrane depolarization along with hyperproduction of ROS (Ref 137). In cancer cells, curcumin was shown to cause mitochondrial disruption leading to ROS production (Ref 138).

## Discussion

The growing global usage of herbal products raises the possibility that plant-based compounds could be a rich source of anti-leishmanials (Ref 139). For any potential compound for use to counter pathogens, it is important to identify a susceptible cellular target in the pathogen, accessible to the drug. Mitochondria are one such organelle that are appropriate targets of multiple drugs (Ref 35) because functionally they maintain cellular homeostasis through energy production and any interference with their operation is detrimental to the cells. The single mitochondrion of the *Leishmania* parasite is a prime target as its functional failure would jeopardize the parasite’s survival ability. In the last decade, many mitochondria-targeted drugs have been synthesized and tested in the mammalian system and some are in clinical trials (Ref 35). Currently, multiple mitochondria-targeted drugs are being tested in mammalian cancer cells (Ref 36, 140) and some of these could be useful as new drugs for leishmaniasis.

Artemisinin and its derivatives have enormous therapeutic value as antimalarials (Ref 141) and have been repurposed for viral infections, autoimmune conditions and cancer (Ref 139). Such efforts indicate the possibility of artemisinin being repurposed as anti-leishmanials because of their efficacy in both models of VL and CL. This has been substantiated by *in vitro* and *in vivo* investigations (Ref 142). Additionally, artemisinin and its derivatives target the mitochondrion of the *Leishmania* making them effective for both forms of the parasite. Chloroquine and its derivatives are used to treat a variety of illnesses, with its efficacy as an anti-leishmanial shown in *in vivo* testing (Ref 143). Coumarins have been thoroughly investigated as medications for different diseases (Ref 87) and are good candidates for anti-leishmanial treatment, especially because they show *in vivo* efficacy against the *Leishmania* parasite. Quinolones are considered to have privileged structures that can be manipulated to generate novel molecules. Quinoline alkaloids show significant anti-leishmanial activities both *in vitro* and *in vivo* and therefore with their background of being tested in humans are good candidates for development as anti-leishmanial drugs. Reported to affect the *Leishmania* mitochondria (Ref 120) quercetin is a good candidate to be considered as a leishmanicidal drug. A summary of the compounds analyzed are detailed in Table 1. In summary, the promising compounds detailed here should be taken up for further investigations for use as anti-leishmanials, as their use has already been tested in the human system.

**Table 1:** Table describing the plants from which the compounds were first prepared, the part of the plants from where the extracts were made, and the targets of the compounds on the parasite mitochondrion

Compound	First derived from	Parent compound	Part of plant	Mitochondrial function targeted
Artemisinin	*Artemisia annua*	Artemisinin	Leaf, root	Inhibits ETC complex I-III and II to III. MMP loss occurs. ROS increase is observed.
Chloroquine	*Cinchona officinalis*	Quinine	Bark	Disruption of ETC complex II and III. There is loss of MMP and ROS increase.
Coumarin	*Dipteryx odorata*	benzo-α-pyrone	Beans Whole plant	ROS increase along with MMP loss is observed.
Quinolone	*Cinchona officinalis*	Nalidixic acid	Bark	Inhibition of ETC with ROS increase
Quercetin	*A. cepa*, *Allium fistulosum*, *Apium graveolens*, *Asparagus officinalis*, *B. oleracea var. sabellica*, *Calamus scipionum*, *Camellia sinensis*, *Capparis spinosa*, *Centella asiatica*, *Coriandrum sativum*, *Daucus carota*, *Emblica officinalis*, *Glycyrrhiza glabra*, *H. perforatum*, *Hypericum hircinum*, *Malus domestica.* *Mangifera indica*, *Momordica charantia*, *Moringa oleifera*, *Morua alba*, *Nasturtium officinale*, *Ocimum sanctum*, *Prunus domestica*, *Solanum nigrum*, *Swertia chirayita*, *Vaccinium oxycoccus*, *Withania somnifera*,	flavonoid group of polyphenols	Seeds, leaves, stems, grains	Loss of ATP, Loss of MMP

## Future directions

The plethora of plant-based compounds and their derivatives with evidence of leishmanicidal activities, offer new opportunities for the field of anti-leishmanials. The possible drugs analyzed above should be taken forward by filling the gaps necessary to satisfy preclinical requirements. Taking lessons from the field of cancer drug development, mitochondria-targeted drugs now being designed for cancer and other diseases (Ref 37, 144) could form a repertoire of possible new anti-leishmanial compounds. Some derivatives of artemisinin synthesized specifically targeting the mitochondria in mammalian cells (Ref 145) have not been tested in the *Leishmania* parasite. These compounds with low toxicity in *in vitro* studies with mammalian cells indicate the suitability of their use as potential agents suitable for pathogen elimination (Ref 145). The chloroquine derivative DNDI-0690 has completed clinical trials for leishmaniasis, further designing of effective drugs based on the structure of this compound has the possibility of obtaining more effective anti-leishmanials. With the high efficacy of the 4-aminoquinoline derivatives against leishmaniasis, further studies are required to generate alternatives to DNDI-0690.

It is expected that researchers will benefit from this review built to detail compounds with *in vivo* efficacy and with effects on the mitochondria. Over the past several years, a significant effort has gone into *in silico* studies where molecular docking has been used to identify potential compounds for anti-leishmanial efficacy, even predicting the structure of their binding sites (Ref 146–149).

A strategy for discovering new uses for approved drugs under investigation for repurposing could be outside the scope of the original medication. One of the major benefits of repurposing is that the drugs have already been tested in humans and have on record full information on their pharmacology, dose, possible toxicity and formulation. This cuts the cost of research and development, it does not require phase I clinical trials in humans and toxicity data are available. Artemisinin, chloroquine and their derivatives known for their efficacy in malaria have been tested in animal studies for leishmaniasis with success. Therefore, the future of drug development against leishmaniasis could firmly advance the findings over the past years on the *in vivo* efficacy with improved drug delivery systems. Other suggestions have been made, for example, the use of structural biology to look for drug targets for leishmaniasis (Ref 150) and, the use of molecular docking and molecular dynamic simulations for anti-leishmanial drug development (Ref 151). Lessons from different fields of research if applied judiciously would provide a huge source of possible plant-based drugs for several diseases. Joint collaborative research in interdisciplinary areas is required to focus on relevant structures of compounds that offer prospects for drug development. Given the complexity of drug development, multiple stakeholders need to be involved. For efficient bench-to-bedside translation, cooperation between the industry and academia is a must as knowledge generation and making formulations suitable for patient use have to go hand in hand. The medical fraternity here plays an important role as they have a complete understanding of diseases. For them to be in the loop not only during preclinical trials but also during drug development is a necessity.
